# Causal loop diagramming the dynamics that shape food environments in Dutch supermarkets

**DOI:** 10.1186/s12916-025-04360-z

**Published:** 2025-10-23

**Authors:** Cédric N. H. Middel, Chiara Colizzi, Wilma Waterlander, S. Coosje Dijkstra, Joline W. J. Beulens, Joreintje D. Mackenbach

**Affiliations:** 1 Epidemiology & Data Science, Amsterdam UMC Location Vrije Universiteit, Amsterdam, the Netherlands; 2 Amsterdam Public Health Research Institute, Amsterdam, the Netherlands; 3 Upstream Team, www.upstreamteam.nl, Amsterdam, the Netherlands; 4 Department of Global Public Health and Bioethics, Julius Centre for Health Sciences and Primary Care, University Medical Centre Utrecht, Utrecht, the Netherlands; 5 Public and Occupational Health, Amsterdam , UMC Location University of Amsterdam, Amsterdam, the Netherlands; 6 Public and Occupational Health, Amsterdam , UMC Location Vrije Universiteit, Amsterdam, the Netherlands

**Keywords:** Commercial determinants of health, Public health, Prevention, Systems thinking, Systems science, Food environment

## Abstract

**Background:**

Food-retail environments are often dominated by unhealthy products, which facilitates unhealthy diets. Limited insight into the factors in the commercial food system that cause this issue makes effective health interventions in retail settings difficult. This study explores the factors and dynamics of the Dutch commercial food system that determine the availability of healthy and unhealthy food in Dutch supermarkets.

**Methods:**

The study developed and analysed a causal loop diagram (CLD) of the factors and dynamics that determine in-store food availability. Data was collected through semi-structured interviews with food system professionals (*n* = 14) and a focus group with academic experts (*n* = 6), based on the leading question ‘what determines the in-store availability of a product?’ Transcripts were qualitatively coded to identify factors and their interactions. These were visualised in a CLD and subsequently examined to identify causal loops and other dynamics.

**Results:**

The CLD revealed a core feedback loop between in-store product availability, sales, and pricing, and how this interacted with consumer behaviour and production and supply. Products that sell well and have large profit margins are made more available. Consumers generally buy products that are tasty and affordable, while products with low production and supply costs have better profit margins. These factors favour abundant availability of products consisting of cheap and highly palatable unhealthy ingredients, leading to a reinforcing feedback loop that disadvantages availability of more costly and perishable healthy products. Competition and innovation further emphasise this dynamic, as producers strive to reduce costs and increase palatability in a race to the bottom. Societal interest in health presents a minor feedback loop that favours healthy products due to their positive public image.

**Conclusions:**

Our findings show that the prevalence of unhealthy products compared to healthy ones is deeply ingrained in the current system dynamics. Policy recommendations include facilitating sustainable corporate governance models, adjusting financial incentives via taxes and subsidies, and enforcing a ‘level playing field’ for healthier business practices.

**Supplementary Information:**

The online version contains supplementary material available at 10.1186/s12916-025-04360-z.

## Background

Unhealthy dietary behaviours present a major global public health challenge [1], and the widespread availability of unhealthy foods in food-retail environments is a known driver of these behaviours [2–4]. Unfortunately, the majority (~ 80%) of products on offer in stores across middle- and high-income countries do not contribute to a healthy diet [5–8]. This past decade, interventions in food retail settings have been a common approach to promote healthier dietary behaviours by adjusting prices, marketing, or product availability [9, 10]. A common strategy in these interventions is to reduce the number of and space for unhealthy products, while increasing them for healthy ones to make healthy products more ‘available’. This has shown promising effects in controlled experiments but limited impact in real-life settings [9–12]. This is likely related to the barriers for implementation and sustainment that these interventions often encounter, such as concerns over costs and profits, resource constraints, or conflicting priorities, leading to faulty implementation or not being maintained [13]. Addressing these barriers is critical for improving the healthfulness of food-retail environments.

Similarities across interventions and contexts regarding the barriers encountered imply common underlying causes [14]. These causes are likely broad in scale and related to the system in which retailers operate. We define this system as a ‘commercial food system’, consisting of *the actors, factors, and dynamics involved in the commercialised production, processing, retail and consumption of food*. This definition is based on the dominant way food chains are organised in Global North countries, where most of the cited literature on food retail interventions and associated barriers come from. Previous studies have shown that barriers for the implementation and sustainment of health interventions in food retail settings (e.g. fears of losing customers or lowering profits) often arise from inherent conflict between this system, which prioritises commercial success, and the interventions in food retail settings, which prioritise health promotion [14–16]. For example, the interventions aim to make fast-selling unhealthy foods less available, while the system incentivises offering such foods through pressure from competition and performance metrics [16]. As such, in order to achieve healthier food-retail environments, it seems necessary that the factors and dynamics in the underlying commercial food system that intervene with efforts to create such environments are addressed.

While there have been studies into food-retail environments, unhealthy diets, and health interventions, from a systems perspective [15, 17, 18], insight into the dynamics that determine the availability of (un)healthy foods in these environments is currently lacking. This makes it difficult to identify what parts of the commercial food system drive the current prevalence of unhealthy foods in food-retail environments, and how these should be changed. Furthermore, this lack of clarity is used by system actors to deflect responsibility [19], sandbagging efforts to change the system. For these reasons, it is vital that the system dynamics that determine healthy and unhealthy foods availability in food-retail environments are unravelled, so that leverage points for change and responsible actors can be identified.

The use of a system dynamics approach, which is specifically designed to map complex dynamics into structured overviews such as a causal loop diagram (CLD), can help to address this gap [15, 20] and has been used previously in relation to food (retail) and unhealthy dietary behaviours [17, 18]. This study will focus on supermarkets, which are a primary site for food purchases, and therefore major dietary point-of-choice in many countries [21]. Thus, we addressed the question ‘*what system dynamics contribute to the availability of healthy and unhealthy foods in supermarkets?*’.

## Methods

Methods were reported in accordance to the consolidated criteria for reporting qualitative research (COREQ) (Supplementary File A) [22].

### Design

We performed a case on supermarkets and their suppliers in the Netherlands. The study followed an iterative approach using causal loop diagramming [23] to map out the factors and dynamics that contribute to the availability of healthy and unhealthy foods in supermarkets. The causal loop diagrams were developed based on interview and focus group data in a process inspired by grounded theory [24]. The study was conducted within a critical realist onto-epistemological paradigm, common within system dynamics [25]. There were two main phases in this study.

**Fig. 1:**
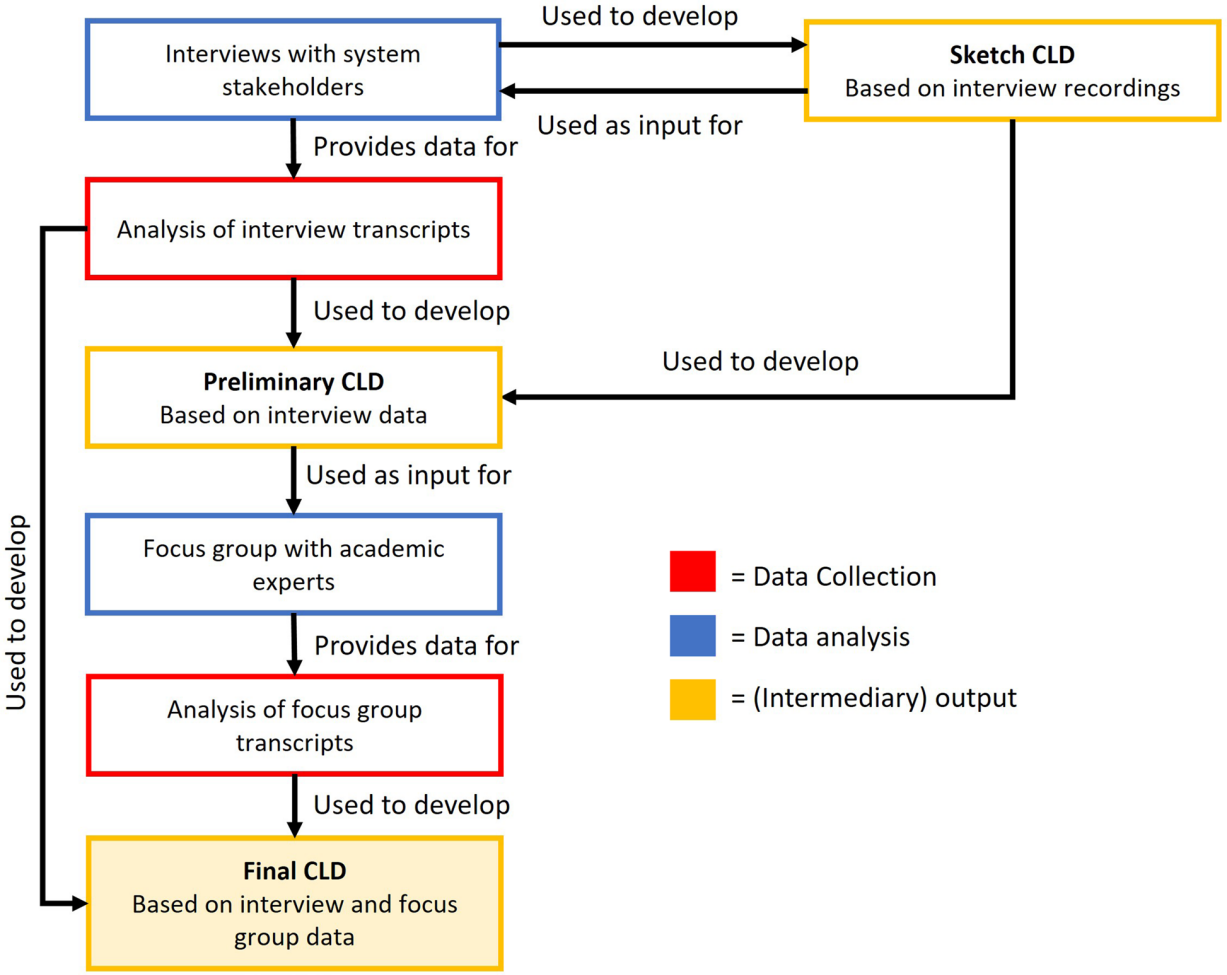
Flowchart of the study process. Shown are the steps taken in the process of this study. Data collection, analysis, and (intermediary) output are colour-coded

In study phase one, commercial food system stakeholders were interviewed to identify relevant dynamics influencing food availability in Dutch supermarkets. We focussed on system stakeholders because, by working within the system, they have knowledge of both the explicit (e.g. rules, processes) and implicit (e.g. values, beliefs) factors and dynamics of the system. The outcomes were integrated into a ‘sketch CLD’ by the researchers, on which subsequent participants were asked to reflect. Individual interviews were used to enable participants to speak freely [26], which they were unlikely to do in a group with direct competitors, and to accommodate their limited availability. Finally, a ‘preliminary CLD’ was developed based on the sketch CLD and the interview data. In study phase two, a focus group was held with academic experts to validate and refine this preliminary CLD. Using both the interview and focus group data, a final CLD version was developed. This process is illustrated in Fig. 1.

### Researcher characteristics

CM (PhD) and CC (MSc) interacted with participants. CM was a male postdoc focussing on food-system drivers of unhealthy diets, particularly in the Netherlands, with previous training in systems thinking and qualitative methods. CM’s contacts in the Dutch food sector from previous studies facilitated participant recruitment, though it may have also introduced bias in participant selection and their responses in interviews. Being a native Dutch speaker and experienced with the topic aided the flow of the interviews. CM’s background in ‘health’ may have biased participants to give socially desirable answers (e.g. emphasise their organisation’s efforts in promoting public health). CC was a female PhD candidate specialising in sustainable food systems and cardiovascular health. She was experienced with qualitative research in the Dutch context. CC was not involved in identifying and recruiting participants. Although CC limited her interaction with participants during interviews, it is possible that CC’s background may have influenced participant responses similarly to CM (inviting socially desirable answers regarding health promotion).

### Context

The study took place in the Netherlands, where supermarket chains dominate the market: supermarket accounted for 77% of total food-related expenses in 2010 [27]. Supermarkets in the Netherlands are relatively small, but densely spread across the country. There are usually multiple per neighbourhood in urban areas and at least one within biking distance in rural areas. Chains offer either a limited range lower prices, or a broader range with higher prices and weekly rotating discounts on certain items. Competition is high (the biggest chains held a 37.1%, 21.1%, and 7.8% market share in 2023) [28] and often based on product prices [15]. In 2021, 79% of the products in Dutch supermarkets are not recommended by the Dutch dietary guidelines (described below) [8], prompting societal calls for (governmental) action. Since then, regulation has remained lacking, with health promotion mostly being voluntary (food-choice logo, product improvement). By 2023, little progress had been made [29]. Healthy food in the Netherlands is defined based on the ‘Wheel of Five’ [30] food pyramid model. It recommends five food groups that should make up the overall diet: fruits and vegetables; bread, grain products, and potatoes; water, tea, and coffee; legumes, nuts, fish, eggs, meats, and dairy (replacements); and oils and fats. The wheel is a dichotomous system where products are either recommended or not. Products that fit within the wheel are considered a healthy choice, while everything outside the wheel is not.

### Participants and sampling

#### Interviews

Participants (Table 1) were recruited from key stakeholder groups, including retail organisations (*n* = 4), packaging and processing organisations (henceforward ‘producers’) (*n* = 5), agricultural organisations (*n* = 2), and consumer organisations (advocacy groups that represent and protect the interests of Dutch consumers) (*n* = 2). Recruitment continued until data saturation (no new major themes for two interviews) was reached. We used purposive sampling. For each stakeholder group, we first identified relevant national and international organisations relevant in the Dutch market. These were contacted via email, addressing pre-existing contacts of the researchers where possible, and otherwise ‘cold calling’ to public email addresses (e.g. marketing or public relations departments). Each contact received one initial email and one reminder (if no response was received). The initial email provided information on the study and requested an interview with a senior individual (assumed to correlate with experience) with ‘professional expertise on product ranges, supply, development, or production’. If the person contacted did not fit this description, we would request the email to be forwarded to a suitable colleague. When someone agreed to participate, a follow-up email was sent, including a detailed description of the study and a consent form. A total of 21 people were contacted, of whom six did not respond, and two did not have time. The remaining 13 participated themselves or referred to a colleague who participated. All participants came from different organisations.

**Table 1:** Participant characteristics. Listed are the participant code, associated stakeholder group, and background

Participant	Stakeholder	Background
Interviews		
R1	Retail	Corporate social responsibility and strategy at supermarket chain A
R2		Product management at supermarket chain B
R3		Product management at supermarket chain C
R4		Business operations and commercial strategy at supermarket chain D
P1	Packaging and processing	Product nutritional (re)formulation at a national dairy brand
P2		Product assortment analysis, and development, innovation, and marketing at a national cake brand
P3		Product assortment analysis, and development, innovation, and marketing at an international dairy brand
P4		Sales at an international packaging and processing company
P5		Founder of a national dairy brand
Ag1	Agriculture	Marketing at a marketing and sales organisation for fruit and vegetables
Ag2		Management at a knowledge platform for agriculture
C1	Consumer	Researcher at a consumer organisation
C2		Researcher at a non-profit for healthier food environments
Focus group		
Ac1	Academia	Expert on health policy, economics, and system dynamics
Ac2		Expert on food systems and food system transformation
Ac3		Expert on policy, food systems, and food system transformation
Ac4		Expert on sustainability, marketing, and consumer behaviour
Ac5		Expert on food value chains and markets
Ac6		Expert on management and entrepreneurship in food retail

#### Focus group

Academic experts (Table 1) were recruited through purposive sampling, based on professional contacts, websites of Dutch universities, and relevant academic publications. Each contact received one initial email, and one reminder (if no response was received). Following a positive reaction to the initial invitation email, the next email included a detailed description of the study and a consent form. A total of eight academic experts were contacted, of whom six agreed to participate, and two did not respond.

#### Researcher-participant relationship

The majority of the participants had no direct relationship with the researchers prior to the study. Two of the retail-aligned participants had participated in previous studies by CM into the food offer in supermarkets, and one of the producer-aligned participants was related to a person CM had collaborated with previously. One of the academic participants had previously collaborated with CM on other projects (but was not involved in this study). At the start of an interview or focus group, the interviewers would briefly introduce themselves, their academic backgrounds and interests, and the goal of the study.

### Ethics

The study design was approved by the ‘BETCHIE’ Ethics Committee of the VU University Faculty of Science (2023–011). We informed all participants about the study design and purpose in advance. The participants all provided written consent for participation in the study and being recorded.

### Data collection

#### Interviews

Interviews were semi-structured and ranged between 30 and 60 min long, conducted between November 2023 and May 2024, in private, by CM and CC, in Dutch, via Microsoft Teams. Each participant was interviewed once. We used a semi-structured interview guide (Supplementary File B) to enable participants to speak in-depth [31]. The interview guide was not pilot tested, but was iterated upon after each interview. Interviews were recorded for transcription purposes and no additional field notes were made.

Because guidance on interviewing approaches for causal loop diagramming is sparse [32], inspiration was drawn from realist evaluation approaches [33]. The first part of the interview was set up to develop insight into the participant’s perception of the problem and its causes, based on ‘theory gleaning’ [33]. This is an approach from realist evaluations that aims to develop insight into how a program (in this case system) functions, by having participants talk about their experiences of working within it, and asking further exploratory and clarifying questions. After introducing the study objective, participants were asked to describe, from their professional perspective, the influences in- and outside their organisation that determine the product offer in supermarkets. This first question aimed to develop a broad overview of all factors involved and thus did not mention ‘healthiness’, to avoid biased responses. Next, probing questions were used to clarify and deepen answers, as well as to inquire after potential ‘causes of causes’, to identify feedback loops. Finally, participants could be asked whether the discussed dynamics affected healthy and unhealthy products the same or differently, and why. When a participant had no additional insights on a specific topic, the interview would move on to the next question, until there were no more topics to discuss.

The second part of the interview was based on ‘theory refinement’ [33]. Participants (except the first) were presented with a ‘sketch CLD’, based on the preceding interviews, and asked about the displayed factors and dynamics, and whether important things were missing. Once the interviewee had no more comments, the interview concluded.

After each interview, CM and CC would replay the recording and add the factors and dynamics described by the interviewee to the sketch CLD. This updated version was emailed to the interviewee for feedback to validate whether the researcher had correctly interpreted the interviewee’s words when updating the CLD. Interviewees were asked to respond if they disagreed with the CLD. One participant requested minor changes, and one responded to express agreement, and 11 did not respond (interpreted as agreement). The updated CLD was then used in the next interview.

#### Focus group

The focus group had the aim to evaluate and refine the ‘preliminary CLD’ developed from the interviews (see Data analysis), based upon Andersen and Richardson’s [34] ‘model refinement script’ for group model building. The focus group was conducted in June 2024, in private, by CM and CC, in Dutch, via Microsoft Teams. It was semi-structured and took 120 min. It was recorded for transcription purposes and no additional field notes were made.

Researchers briefly introduced the study to the participants and presented them with the preliminary CLD (Supplementary File C). This CLD was presented in sections, based on the number of links between factors (indicating strong interconnections). For each section, participants were asked whether they agreed with the contents of the CLD, and what they would add, remove, or adjust based on their expertise. After every section had been discussed, participants provided their final remarks, and the focus group was concluded.

### Data analysis

Data was analysed by CM and CC following an inductive approach, adapted from Kim and Andersen’s [35] method for generating causal maps. This approach integrates realist and relativist perspectives, in line with this study’s critical realist approach. Coding was done in MAXQDA [36].

#### Interviews

After concluding the interviews, an initial codebook (Supplementary File D) was developed based on the sketch CLD developed during the interviews. Next, the interview transcripts were coded semi-openly, adding new codes where needed [35]. CM and CC each coded half of the interviews and reviewed each other’s coding. This saved time while still allowing for triangulation between the researchers.

Once coding was finished, CM visualised the coded variables and causal relations in MAXQDA [35]. Arrows were marked as positive or negative causal relations based on the underlying data. Once all causal codes were represented by arrows, the resulting visualisation was the first draft of the preliminary CLD. CM and CC critically reviewed the definitions and relevance of variables and arrows in the preliminary CLD and compared these to the sketch CLD to identify potentially missed factors. Next, the preliminary CLD was discussed with all authors, to identify important dynamics and feedback loops (marked as reinforcing (R) or balancing (B)) and receive additional input. Based on these discussions, further adjustments were made, and the preliminary CLD was remade in Vensim software [37] to improve its visualisation. This version served as input for the focus group: CM and CC divided the CLD into sections based on which variables were most closely connected, for ease of presentation. This roughly corresponded to (1) retail, (2) production and innovation, (3) consumer characteristics and trends, and (4) societal trends and marketing. Furthermore, they selected what were considered the main dynamics within the CLD and visualised these in a simplified figure.

#### Focus group

The transcript of the focus group was analysed via the same approach described above, using the codes already developed for the interviews. Where appropriate due to new insights from this data, the coding of the interviews was also revisited. Next, the CLD was further developed using the combined data from the interviews and focus groups. Next, the CLD was re-examined to evaluate previously identified and identify new feedback loops. The CLD was pruned by removing variables that were not directly vital to understanding these feedback loops, or presented exogenous influences on the described system (e.g. only had a connection with one variable). Notable dynamics that were not part of a loop but did pose a substantial influence were also labelled (D). The CLD was presented to and discussed with all co-authors for final adjustments. The resulting product is presented in our Results section.

## Results

**Fig. 2:**
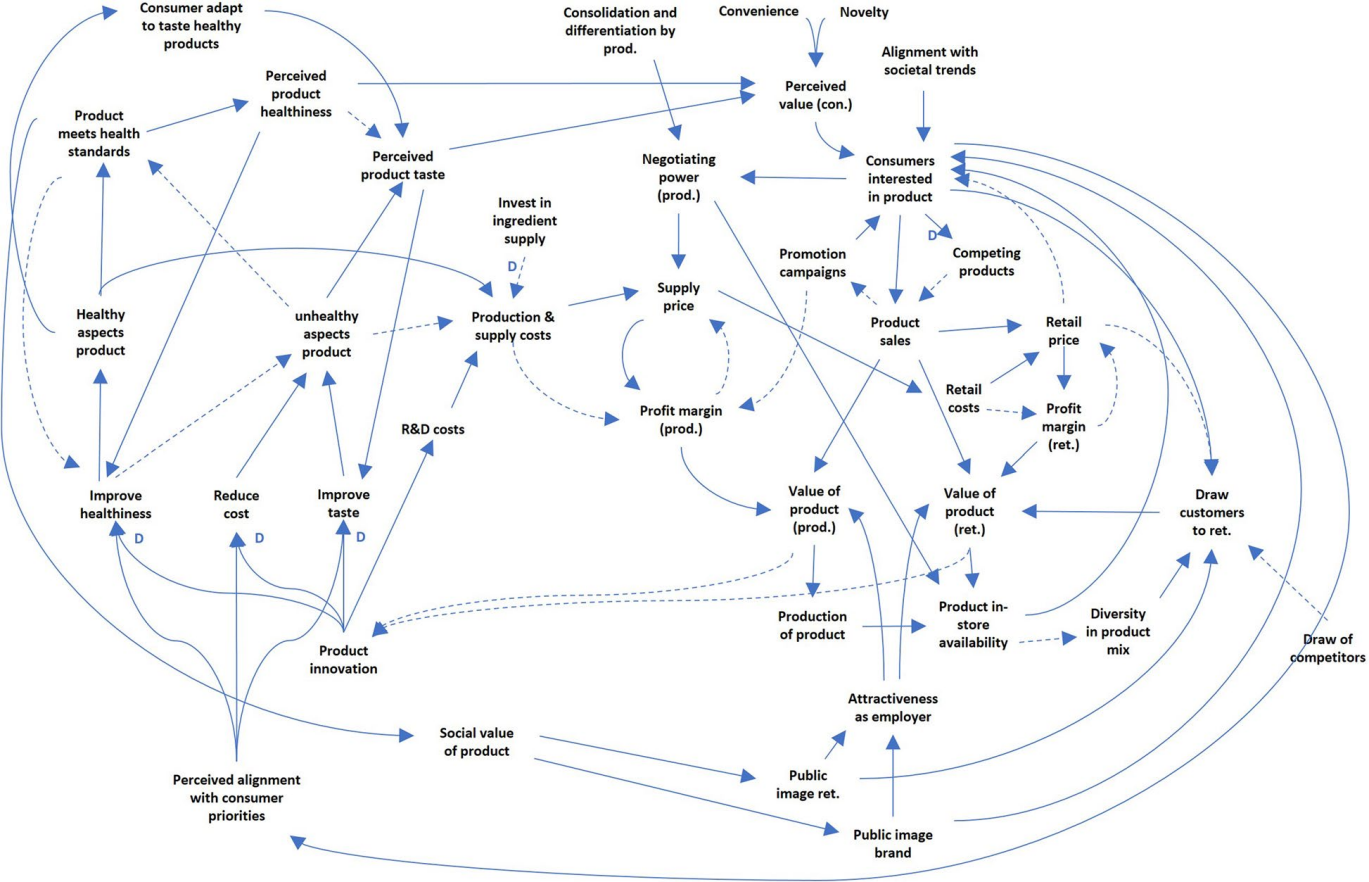
The complete CLD. Straight lines show a synergistic interaction, dashed lines show an inversed interaction. Lines marked ‘D’ have a delayed effect; prod. = producer; ret. = retailer; con. = consumer

The following sections present the system dynamics that play a role in the in-store availability between healthy and unhealthy products. First, the complete CLD is shown in Fig. 2. This CLD is split into two subsystems, representing (1) the subsystem of *supply, retail, and consumption* and (2) the subsystem of *production and innovation*. Both subsystems will be discussed in a separate section below.

### The supply, retail, and consumption subsystem

**Fig. 3:**
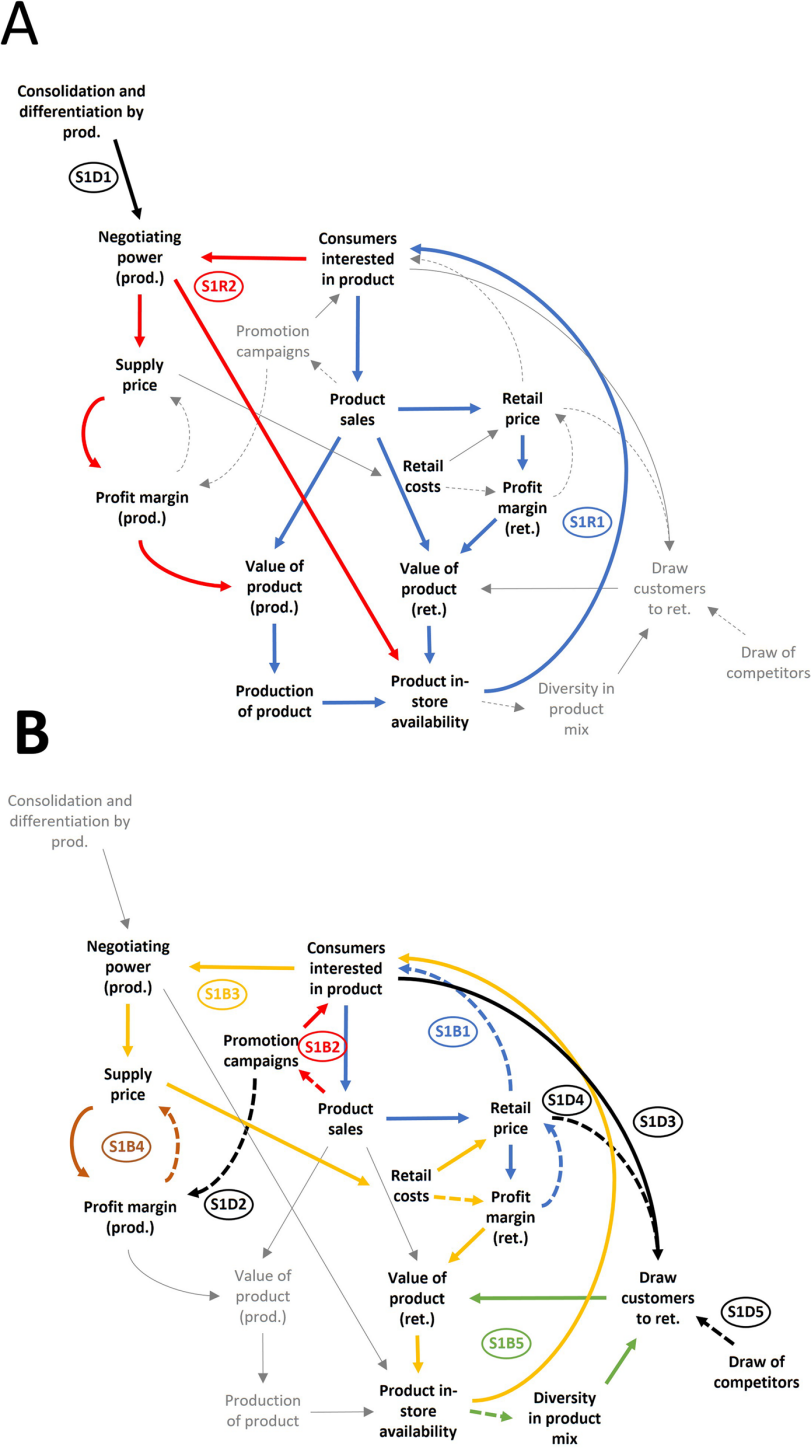
**A** and **B** The *supply, retail and consumption* subsystem (subsystem 1). Shown are the feedback loops and dynamics related to the supply, retail, and consumption of food products. For readability, these loops and dynamics are highlighted in distinct colours and across two images (**A** and **B**). Feedback loops and notable dynamics are labelled and summarised in Table 2. Straight line = synergistic interaction; dashed line = inversed interaction; D = delayed interaction; prod. = producer; ret. = retailer; con. = consumer

The *supply, retail, and consumption subsystem* (Fig. 3A and B) contains the core feedback loops and dynamics in the CLD that drive the system. These loops concern the decision making in supermarkets regarding product availability and pricing, and the interaction of this decision making with consumer behaviours and the product suppliers. The supplier role can be played by intermediaries between producers and retailers, or the producers themselves. Henceforward, we discuss suppliers and producers as a single actor group producers. An overview of the identified loops and dynamics is given in Table 2.

**Table 2:** The *supply, retail, and consumption* subsystem (subsystem 1). This table summarises the feedback loops in the supply, retail, and consumption subsystem. The label column corresponds with Fig. 3

Label	Feedback loop	Mechanism
Reinforcing loops		
S1R1	Consumers interested in product + > product sales • + > value of product (ret.) + > product in-store availability + > consumers interested in product • + > retail price + > profit margin (ret.) + > value of product (ret.) + > product in-store availability + > consumers interested in product • + > value of product (prod.) + > production of product + > product in-store availability + > consumers interested in product	Products which generate high consumer interest sell well. This makes them commercially valuable to retailers and producers. Such products will be offered more in stores, and this availability incites further interest. Inversely, unpopular products are less valuable and thus become less available over time until they are phased out
S1R2	Consumers interested in product + > negotiating power (prod.) • + > product in-store availability + > consumers interested in product • + > supply price + > profit margin (prod.) + > value of product (prod.) + > production of product + > product in-store availability + > consumers interested in product	Consumer interest in a product strengthens producers’ negotiation position versus the retailer. This enables them to gain better profit margin on the product and to negotiate more space for the product. These result in greater in-store availability of the product, which further raises consumer interest
Balancing loops		
S1B1	Product sales + > retail price—> consumers interested in product + > product sales Retail price + > profit margin (ret.)—> retail price	Retailers balance profit margins and sales against each other. When sales are low, retailers can reduce the retail price to generate more consumer interest and sales. This lowers the profit margin. When the profit margin is low, retailers can increase the price, which then lowers the sales
S1B2	Product sales—> promotion campaigns + > consumers interested in product + > product sales	When the sales of a product are too low, producers can use promotion campaigns to boost its popularity temporarily until the sales have risen enough
S1B3	Consumers interested in product + > negotiating power (prod.) + > supply price +> retail costs • + > retail price—> consumers interested in product •—> profit margin (ret.) + > value of product (ret.) + > product in-store availability + > consumers interested in product	Producers of popular products can negotiate higher prices, up to a limit. Supply prices can inflate retail prices which may deter consumers, or detract from the retailer’s profit margin, making the product unattractive to offer in stores. Both result in reduced availability and interest in the product, reducing the negotiating power of the producer
S1B4	Supply price + > profit margin (prod.)—> supply price	Producers base their supply price on a minimal profit margin. When the margin becomes too narrow, prices can be raised, and when the margin is sufficient there is space to lower prices
S1B5	Product in-store availability—> diversity in product mix + > draw consumers to ret. + > value of product (ret.) + > product in-store availability	Retailers aim for a varied range of products to draw a wide range of consumers. When one producer becomes too dominant in the product mix, retailers aim to offer other products instead
Notable dynamics		
S1D1	Consolidation and differentiation by producer + > negotiating power (prod.)	When production is consolidated under one producer, or a product has differentiating qualities, this increases the producers’ market share and negotiating power
S1D2	Promotion campaigns—> profit margin (prod.)	The costs of promotion campaigns detract from profit margins. Therefore, promotion campaigns are mostly used as a temporary boost, after which the product needs to perform independently
S1D3	Consumers interested in product + > draw consumers to ret.	Products that are popular attract consumers to a store
S1D4	Retail price—> draw consumers to ret	Offering too many high-priced items can give a retailer a reputation for being expensive, which can deter consumers
S1D5	Draw of competitors—> draw consumers to ret	A retailer’s ability to attract consumers is in direct comparison to the attractiveness of their competitors

+ >, positive interaction;—>, negative interaction; *D*, delayed interaction; •, alternative paths; *prod.*, producer; *ret.*, retailer; *con.*, consumer

The *supply, retail, and consumption subsystem* (Fig. 3A and B) contains two reinforcing and five balancing feedback loops, and five other dynamics of note (Table 2). The core mechanism of this subsystem consists of two reinforcing loops. The first of these (S1R1; Fig. 3A) describes how product availability and sales performance positively reinforce each other through several parallel paths. Retailers and producers generally give more space to and produce more of products that have commercial value (profitability and/or sales). As such, those products become more available in the retail environment and visible and accessible to consumers, raising interest in and sales of the product, or enabling price raises to benefit profit margins. The end-result is that commercially strong products increasingly dominate the retail environment, which facilitates their performance, whereas underperformers are slowly phased out by the retailer or producer.

The second reinforcing loop (S1R2; Fig. 3A) illustrates how powerful producers can dominate product ranges. When a product is popular among consumers, its producer gains negotiating power versus the retailer. Unique products or a strong position in the market (e.g. international premium brands) similarly enable negotiating power (S1D1; Fig. 3A). Powerful producers can negotiate higher supply prices to retailers, making the product more profitable to produce, or more product facings in the store, with the resulting consumer interest raising sales and the producer’s negotiating power.

The first balancing loop (S1B1; Fig. 3B) describes a balancing mechanism between retail sales and profit margins. Sales will generally be highest when a product is priced low, but this comes at the cost of its profit margin. Alternatively, profit margins higher prices grant greater profit margins, but negatively impact sales. The retail price here serves as a lever to optimise the balance between sales and profit margins for maximal commercial value. This can involve temporary (price promotions), or long-term (lasting price changes) strategies.

Loop S1B2 (Fig. 3B) illustrates that producers (in particular major brands) can use out-of-store promotion campaigns (e.g. commercials, advertisements, sponsoring events) to increase a product’s visibility to incite consumer interest and increase sales. Generally, these campaigns are used when sales are (expected to be) lower than desired and only employed for a limited time because promotion campaigns pose a substantial cost to the producer S1D2 (Fig. 3B).

Loop S1B3 (Fig. 3B) describes a balancing mechanism to the negotiating power of producers of popular products. Although powerful producers can negotiate higher prices, there is a ceiling where this becomes detrimental to sales, or the retailer’s profit margin becomes too narrow. In both cases, the product loses its value to the retailer, which reduces the negotiating power of the producer. Therefore, retailers and producers are incentivised to seek a mutually profitable price point. Another notable loop S1B4 (Fig. 3B) shows how producers, similar to retailers, aim for a minimum profit margin and will negotiate a higher supply price if it becomes too low, while allowing for lower prices (to boost sales) if it is high enough.

The final loop, S1B5 (Fig. 3B), explains that retailers aim for a diverse product-mix, to be able to serve and attract customers with different preferences. When one product (type) dominates the assortment, this lowers the product-mix diversity that is needed to attract a diverse range of consumers. Therefore, retailers choose to add products that improve diversity, while reducing products that are already dominant in the product-mix. This mechanism counteracts the tendency of popular products to increase in availability.

Several dynamics act upon the discussed feedback loops. S1D3 (Fig. 3B) shows how popular products attract consumers to a store (especially when competitors do not offer them), which grants these products secondary value to the retailer, as attracted consumers often buy multiple products. This explains why stores will offer popular but relatively unprofitable products (often major brands such as Coca Cola and Heineken). S1D4 (Fig. 3B) shows how lower product prices can attract, or higher prices can repulse consumers, adding secondary value to products with a low price, despite lower profit margins. Retailer price rankings help consumers to be aware of price differences. Finally, S1D5 (Fig. 3B) describes the ongoing competition between retailers over consumers. This provides an ongoing incentive for retailers to ensure that they remain attractive (e.g. low prices, varied and popular products). Notably, different retailers use different strategies. Some have optimised their business model around limited diversity but lower prices, whereas other have aimed for greater variety with higher prices.

### The production and innovation subsystem

**Fig. 4:**
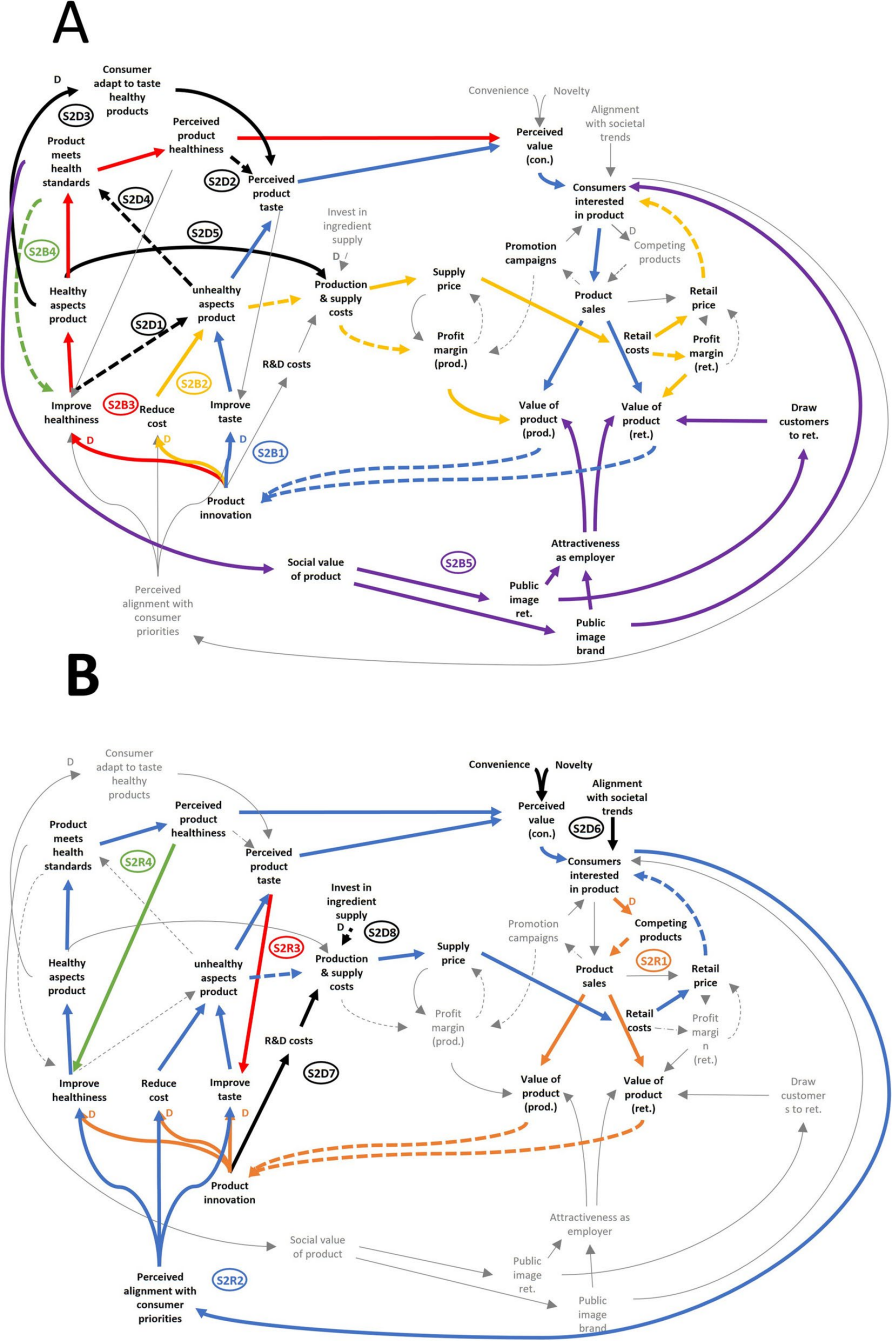
**A** and **B** The *production and innovation* subsystem (subsystem 2). Shown are the feedback loops and dynamics related to the production and innovation of food products. For readability, these loops and dynamics are highlighted in distinct colours and across two images (**A** and **B**). Feedback loops and notable dynamics are labelled and summarised in Table 3. Straight line = synergistic interaction; dashed line = inversed interaction; D = delayed interaction; prod. = producer; ret. = retailer; con. = consumer

The second subsystem concerns *production and innovation* (Fig. 4A and B). This part of the CLD describes the feedback loops and dynamics through which (new) products are developed and improved, and how this impacts the core loops described in the previous section. An overview of the loops and dynamics is given in Table 3.

**Table 3:** The feedback loops of the *production and innovation* subsystem (subsystem 2). This table summarises the feedback loops of the *production and innovation* subsystem. The label column corresponds with Fig. 4

Label	Feedback loop	Mechanism
Reinforcing loops		
S2R1	Consumers interested in product + D > competing products—> product sales + > value of product (prod.)/(ret.)—> product innovation • + D > improve taste + > unhealthy aspects product + > perceived product taste + > perceived value (con.) + > consumers interested in product • + D > reduce cost + > unhealthy aspects product—> production & supply costs + > supply price + > retail costs + > retail price—> consumers interested in product • + D > improve healthiness + > healthy aspects product + > product meets health standards + > perceived product healthiness + > perceived value (con.) + > consumers interested in product • + D > improve healthiness +> healthy aspects product +> product meets health standards + > perceived product healthiness + > perceived value (con.) + > consumers interested in product	When a product is/becomes popular, competitors often develop similar products to compete over market share with the original, detracting from its sales. This stimulates ongoing innovation to remain attractive to consumers
S2R2	Consumers interested in product + > perceived alignment with consumer priorities • + > improve taste + > unhealthy aspects product + > perceived product taste + > perceived value (con.) + > consumers interested in product • + > reduce cost + > unhealthy aspects product—> production & supply costs + > supply price + > retail costs + > retail price—> consumers interested in product • + > improve healthiness + > healthy aspects product + > product meets health standards + > perceived product healthiness + > perceived value (con.) + > consumers interested in product	Producers and retailers use market research and experience to determine what characteristic of a product are most important to consumers. Innovation will focus on that characteristic
S2R3	Perceived product taste + > improve taste + > unhealthy aspects product + > perceived product taste	Innovation on products perceived as tasty by consumers will prioritise taste
S2R4	Perceived product healthiness + > improve healthiness + > healthy aspects product + > product meets health standards + > perceived product healthiness	Innovation on products perceived as healthy by consumers will prioritise healthiness
Balancing loops		
S2B1	Product innovation + D > improve taste + > unhealthy aspects product + > perceived product taste + > perceived value (con.) + > consumers interested in product + > product sales + > value of product (prod.)/(ret.)—> product innovation	Product innovation can improve product taste, by increasing unhealthy aspects. This makes the product more attractive, raising sales and reducing the incentive for further innovation
S2B2	Product innovation + D > reduce cost + > unhealthy aspects product—> production & supply costs • —> profit margin (prod. & sup.) + > value of product (prod.)—> product innovation • + > supply price + > retail costs—> profit margin (ret.) + > value of product (ret.)—> product innovation • + > supply price + > retail costs + > retail price—> consumers interested in product + > product sales + > value of product (prod.)/(ret.)—> product innovation	Product innovation can reduce production and supply costs, by increasing unhealthy aspects. This raises the profit margin or lowers the retail price (and thus sales), increasing commercial value and reducing the incentive for further innovation
S2B3	Product innovation + D > improve healthiness • + > healthy aspects product + > •—> unhealthy aspects product—> Product meets health standards + > perceived product healthiness + > perceived value (con.) + > consumers interested in product + > product sales + > value of product (prod.)/(ret.)—> product innovation	Product innovation can improve the (perceived) healthiness of a product, increasing healthy aspects and reducing unhealthy ones, until a legal standard is met, allowing the product to be presented as ‘healthy’. This will raise sales of the product, reducing the incentive for further innovation
S2B4	Improve healthiness + > healthy aspects product + > product meets health standards—> improve healthiness	Health improvements in products have diminishing returns once a legal cutoff point has been reached, as the next cutoff then takes substantial additional improvements
S2B5	Product innovation + D > improve healthiness + > healthy aspects product + > product meets health standards + > social value of product • + > public image ret. + > draw consumers to ret. + > value of product (ret.)—> product innovation • + > public image ret. + > attractiveness as employer + > value of product (prod.)/(ret.)—> product innovation • + > public image brand + > consumers interested in product + > product sales + > value of product (prod.)/(ret.)—> product innovation • + > public image brand + > attractiveness as employer + > value of product (prod.)/(ret.)—> product innovation	Products legally presentable as healthy can facilitate a positive public image for the retailer and producer, drawing customers to the store and brand, increasing overall sales, thus reducing the need for further innovation
Notable dynamics		
S2D1	Improve healthiness—> unhealthy aspects product + > perceived product taste	Improvements in the healthiness of a product often come at the cost of taste
S2D2	Perceived product healthiness—> perceived product taste	Consumers associate products presented as healthy with worse taste
S2D3	Healthy aspects product + D > consumer adapt to taste healthy product + > perceived product taste	Through exposure, consumers’ taste becomes accustomed to healthier products
S2D4	Unhealthy aspects product—> product meets health standards	Improvements in taste or cost often make a product less healthy
S2D5	Healthy aspects product + > production & supply costs	The healthy aspects of a product can add to its production or supply costs
S2D6	1) Retail price—> perceived value (con.) + > consumers interested in product 2) Perceived product taste/perceived product healthiness/convenience/novelty + > perceived value (con.) + > consumers interested in product 3) Alignment with societal trends + > consumers interested in product	Consumer interest is based on price, taste, healthiness, convenience, novelty, and alignment with current societal trends
S2D7	Product innovation + > R&D costs + > production & supply costs	Product innovation carries costs, which need to be compensated by improved sales and/or profitability of the product
S2D8	Invest in ingredient supply -D > production & supply costs	Producers invest in reliable suppliers for (newly needed) ingredients, which in time reduces production costs

+ >, positive interaction;—>, negative interaction; *D*, delayed interaction; •, alternative paths; *prod.*, producer; *ret.*, retailer; *con.*, consumer

The *production and innovation subsystem* contains four reinforcing and five balancing feedback loops, and eight other dynamics of note (Table 3). Three balancing loops form the core of this subsystem and will be explained first. S2B1, S2B2, and S2B3 (Fig. 4A) illustrate three main approaches to product innovation (meaning improvement or replacement with a superior alternative). Product innovation is generally applied to raise the commercial value of products whose value is perceived as (too) low, because of disappointing sales, profit margins, or ability to attract customers. Producers can initiate it independently or be stimulated by retailers.

S2B1 (Fig. 4A) describes the first innovation approach, which is to improve the taste of a product to increase its attraction. This often amplifies the unhealthy aspects of the product (e.g. sugar or salt content) as such ingredients are highly palatable. Improved taste is perceived as valuable by consumers and thus incites their interest. In turn, this increases sales and the commercial value of the product, which reduces further pressure for innovation.

S2B2 (Fig. 4A) describes the second approach, aimed at reducing production, supply, and retail costs. This is done by adding preservatives or replacing ingredients with cheaper versions, which often amplifies the unhealthy aspects of the product. This reduces costs for producers and retailers, giving space for greater profit margins, or lower retail prices, which producers and retailers indicated as the most important driver of consumer interest and sales. As a result of this benefit to margins and/or sales, the increased commercial value reduces further pressure for innovation.

S2B3 (Fig. 4A) describes the third approach, aimed at improving product healthiness. This is done by improving healthy aspects (e.g. adding fibres) or reducing unhealthy ones (e.g. reducing salt), until a certain goal is met, often based on legal standards for health-related statements or logos (e.g. ‘low in sugar’, or a healthy-choice logo). This enables the product to be presented as ‘healthy’ to consumers (which does not guarantee it being healthy). Such products are more likely to incite consumer interest. This raises sales and the increased commercial value reduces further pressure for innovation. Notably, producers and retailers perceived taste and price to be more important to most consumers, and therefore more effective approaches in many cases. Furthermore, health improvement has diminishing returns (S2B4; Fig. 4A): when a cutoff point for a health-related statements or logo is, reaching the next level is often far away, or there is none. This disincentivises further innovation in this direction.

Despite S2B1, S2B2, and S2B3 being balancing loops, innovation is often ongoing. One of the reasons for this is inter-product competition (S2R1; Fig. 4B). When a product becomes popular, this will be noticed by competitors (which can include retailers’ own brands), who will attempt to develop products that can compete over that market segment. When these products enter the market, they will detract from the sales of the original product, reducing its sales and commercial value. This incentivises further innovation to stay ahead of the competition and maintain consumer interest. One participant described this dynamic where competitors develop increasingly attractive but unhealthy products as a ‘race to the bottom’.

Notably, there is friction between health-based innovation (S2B2) and taste and cost-based innovation (S2B1 and S2B3). Dynamic S2D1 (Fig. 4A) illustrates that improving healthiness can negatively affect taste and may cause products to become less attractive. Furthermore (S2D2; Fig. 4A), consumers generally expect products presented as healthier to be less tasty, detracting from the value of health as a means to incite interest in a product. However (S2D3; Fig. 4A), consumer do eventually adapt to the taste of ‘less tasty’ healthier products. Inversely, S2D4 (Fig. 4A) describes how improving taste or reducing cost can result in a product no longer being allowed to use health-related statements or logos, which would harm its attractiveness. Finally, S2D5 (Fig. 4A) shows that healthier products (e.g. few preservatives, fresh ingredients) often have higher production or supply costs (e.g. more expensive ingredients, shorter shelf-life), which negatively affect prices or profit margins.

Because of the aforementioned friction, product innovation generally prioritises one approach over others. Loop S2R2 (Fig. 4B) describes how, based on market research, retailers and producers estimate which product qualities will most likely incite consumer interest, and this informs which innovation approach is prioritised. If the chosen approach raises interest in the product, the perception that this approach is effective is reinforced, making it more likely to be used in the future. Alternatively, if the product does not become (more) popular, this incentivises the use of other approaches in the future.

This prioritisation is highly dependent on how consumers value different product qualities (S2D6; Fig. 4B). Our participants perceived cost, taste, convenience, novelty, and healthiness to be the main qualities of importance. Out of these, cost was agreed to be the most important, followed by taste. This contrasted heavily with the growing societal interest in health that many participants perceived, which retailers and producers described as a ‘citizen-consumer paradox’: society can consider something important, but in their role of consumer most individuals tend to make different choices.

Another factor in innovation prioritisation is the image of a product (S2R3 and S2R4; Fig. 4B). Consumers usually perceive products in a certain role, which changes what they value about them. As such, for products perceived as tasty, the priority is to innovate upon taste, while for products perceived as healthy, the priority is to innovate upon healthiness.

Despite its commercial disadvantages, health-focussed innovation can be valuable, by boosting public image (S2B5; Fig. 4A). Supermarket rankings in the media often include how ‘healthy’ the product offer is. Ranking high, by offering healthy products, can boost the public image of a retailer and thus attract more customers. Similarly, healthy products can reflect positively on their associated brand and thus attract more consumers to buy products from that brand. In both cases, this indirectly adds commercial value and reduces the need to innovate or reduce availability. Retailers and producers also indicated that a positive public image helps attract motivated employees, which adds on to the value of products that boost this image.

## Discussion

**Fig. 5:**
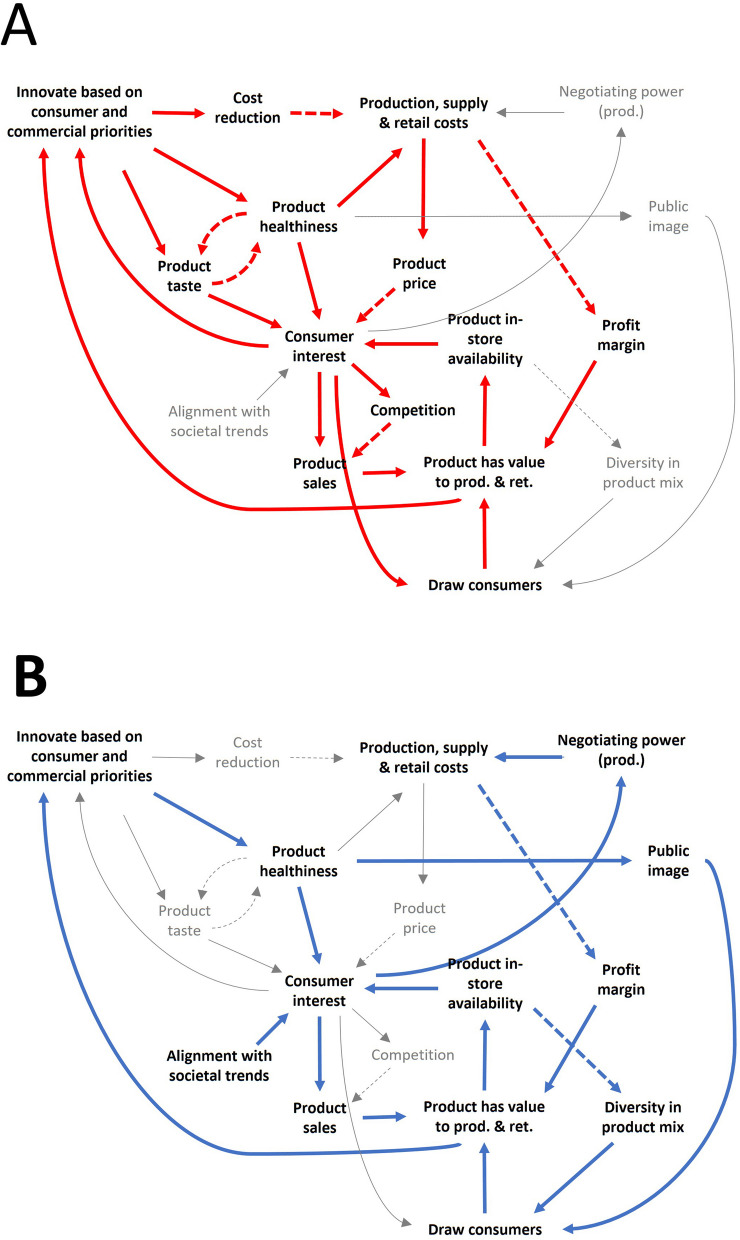
Summary of dynamics driving product availability. Shown is a simplified CLD of notable drivers of the availability of unhealthy products (highlighted in red, in **A**) and healthy products (highlighted blue in **B**) in supermarkets. Straight lines show a synergistic interaction, dashed lines show an inversed interaction. prod. = producer; ret. = retailer

Using a system dynamics approach, we identified the main dynamics underlying the availability of healthy and unhealthy food products in supermarkets as summarised in Fig. 5.

A key quality of commercially successful food products is the ability to incite consumer interest. This is most strongly driven by low cost and good taste, with health being less important. As a result, unhealthy products, which are generally cheaper and tastier, tend to outperform healthy ones in terms of sales, leading to greater availability in supermarkets due to higher commercial value. Likewise, product innovation is mostly used to further increase product value through tastiness and reduced costs, because these characteristics are most likely to result in commercial success. Healthiness in contrast is commercially more challenging to leverage, because it raises cost and potentially negatively influences taste. This reinforces the abundance of unhealthy, cheap, and tasty products in food-retail environments. Competition over consumers’ limited budgets further disincentivises the development and innovation of healthier products, because the first producer to do so takes a commercial risk (healthiness is less likely to lead to commercial success), while competitors (other producers and retailers’ private labels) can simply copy ‘what works’ (a so-called first-mover disadvantage). The same dynamic disincentivises retailers from replacing unhealthy products with healthier products, at the risk of consumers moving to competitors who only copied the ‘popular’ replacements. The negotiating power of producers plays a balancing role: Powerful producers can negotiate higher supply prices, making the product less profitable for retailers, although in practice, such products would also likely have greater sales volumes and attract customers. In reverse, less powerful producers would likely offer products that are unlikely to become substantially more popular for one reason or another (e.g. vegetables).

There are also several dynamics that benefit the availability of healthy products. (i) Offering healthy products available can attract consumers interested in healthiness (a market that has been growing recently [38]. (ii) Offering healthy products can boost the image of the producer or retailer among consumers that value this, although this effect seems highly sensitive to the current public discourse (e.g. low sugar options, when sugar dominates the discourse) and can easily flip to scepticism [39]. (iii) Fresh healthy products have relatively high profit margins for Dutch retailers due to the low negotiating power of their producers (according to our participants), although the literature on this subject is lacking. (iv) The popularity of healthy foods can rise due to social trends [40]. However, the current low availability of healthy products in food-retail environments [5–8] illustrates that dynamics that favour unhealthy products clearly outweigh those that benefit healthy ones. This begs the question what can be done to address this issue?

### Changing the system: where to begin?

**Fig. 6:**
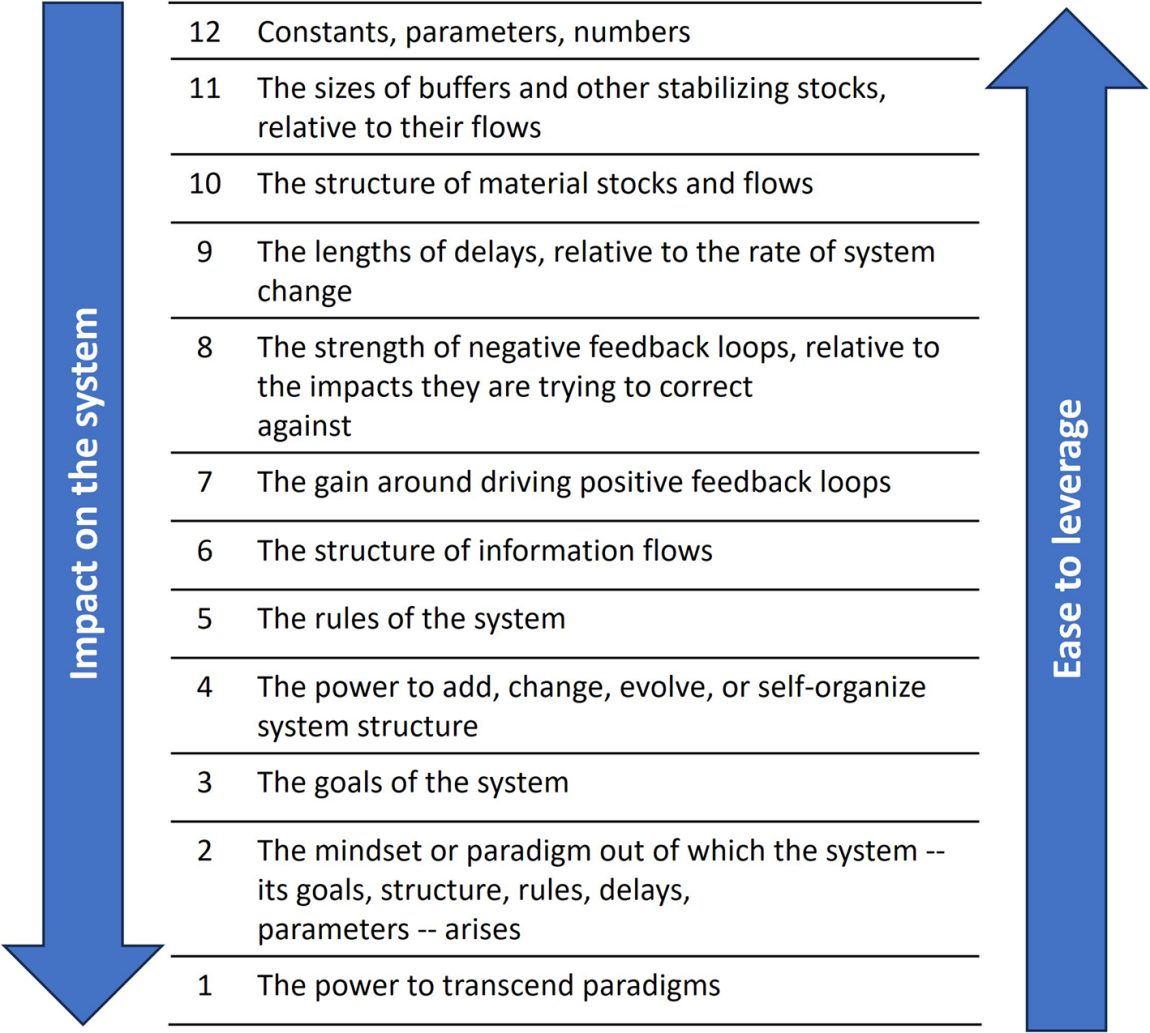
The framework of leverage points for system change, based on Meadows [41]. Leverage points are numbered from high to low, with higher points being more effective at driving system change, but also more difficult to leverage due to resistance from within the system

Below we discuss potential leverage points for changing the system dynamics of unhealthy food product availability through the lens of Meadows’ framework of leverage points for system change [41, 42]. This framework describes 12 levels of leverage points in systems for achieving change (see Fig. 6). Higher levels are more difficult to leverage but change the system on a fundamental level. Lower levels are easier to leverage but only have limited impact on the system. Lower-level leverage points can also be used to then influence higher level ones. Below we will discuss several potential leverage points that have emerged from our findings.

#### Goals of the system

The highest leverage point that stands out is the dominant role of profitability, or ‘commercial value’, in the system, which can be regarded as its most important goal (*leverage point 3*) [41]. This has also been discussed by Swinburn et al. who explained how maximising profits via ever increasing consumption and externalisation of costs is inherently at odds with healthy and moderate consumption [43]. This goal exemplifies the difficulty in creating healthier food-retail environments, as health promotion is diametrically opposed to the goals of commercial food systems. Therefore, a fundamental shift is needed in how business in the food sector is conducted, without the current emphasis on profit and growth. Inspiration could be drawn from alternative economic paradigms, such as postgrowth or sufficiency [44, 45], that are proposed for food systems [46]. Inspiration can be drawn from small-scale examples in the energy sector [47]. However, broad adoption should be a topic for future research in itself, as it would likely require a massive shift in (inter)national economic models and will likely face resistance from the beneficiaries of the current capitalist model.

#### Rules of the system

One way to work towards changing the goals of the system is by using the lower situated leverage point of the ‘rules’ of the system (*leverage point 5*) [41], such as governance models. The observed emphasis on profitability is typical for the ‘shareholder value’ mode of corporate governance from the USA, in which publicly held corporations are beholden to their shareholders and primarily exist to maximise value for them [48, 49]. This logically leads to the exploitation (e.g. producing addictive or unsustainable products) of other stakeholders (e.g. customers, environment) to the benefit of shareholders, who are insulated from legal backlash [49]. In the Netherlands, corporate governance follows the ‘stakeholder value’ model, which in theory aims to maximise value for all stakeholders (e.g. shareholders, employees, other impacted groups), to avoid the issues of the shareholder value model [49, 50]. However, due to lack of accountability and influence, heterogeneity of stakeholder interests, and stakeholder and shareholder interests often being opposed, there remains space for externalities, especially those impacting distant (e.g. future) stakeholders [49, 50]. The issues with such governance structures become clear when shareholder value is threatened, for example the profitability-related barriers encountered by health interventions in supermarkets [14]. To change the commercial food system rules, alternative models of corporate governance that effectively prescribe sustainable practices could be explored, such as steward ownership (a model in which control and financial rights are separated) [51].

#### Reinforcing feedback loops

Another possible step would be to address the reinforcing loops in the system (*leverage point 7*) [41]. An important loop is the competition and the availability of unhealthy products. Several participants indicated that their organisation makes an effort to produce at least some healthy products, for moral reasons. Yet, they prioritise the most profitable and attractive (unhealthy) products due to competition pressures, resulting in a ‘race to the bottom’. This observation is supported by the literature: A previous study found that retailers often argued for a ‘level playing field’ (meaning policies regulating competition on unhealthy products) before they could commit to changing their product prices and offer, because they feared losing customers to not-health-promoting competitors [52], an expectation supported by the literature [53]. Furthermore, evidence on whether healthy product promotion can be a viable business strategy in current food retail markets is mixed, according to a systematic review on the commercial outcomes of common ‘healthy food retail strategies’ [54]. Based on these observations, it seems clear that some form of market regulation is required in order to make healthier food environments (commercially) viable. For inspiration, we can look at what is proposed for alcohol, such as limiting where products are offered, or when and how they are promoted [55]. In the context of our findings, regulating where or how many unhealthy products can be offered by retailers would reduce their availability and its reinforcing effect on consumer interest. Similarly, limiting promotion opportunities would weaken the impact of promotion campaigns on consumer interest. Other strategies could focus on prices and profit margins (discussed further below) to make products less desirable for consumers or profitable for producers or retailers, which feeds back into the overall value of a product for retailers and producers.

When considering the formulation of such regulatory approaches, an interesting observation is that the food sector seems to support stronger market regulations [52, 56]. However, when the food sector is involved in formulating such policies, it often seems to result in plans for regulation being diluted or delayed [57, 58]. This casts doubt on their sincerity and begs the question whether they should be involved in the formulation of such policies at all.

#### Lower-level leverage points

Finally, there are several lower-level leverage points that, despite limited impact, may be useful as ‘small wins’ to build momentum for higher-level leverage points [59]. One of these is by strengthening the balancing feedback loops related to societal interest in health (*leverage point 8*). Raising food label literacy could do this by enhancing health consciousness [60] and arming consumers against deceitful marketing practices such as ‘health washing’ [61]. Holistic and warning front-of-pack labels seem most effective at accurately informing consumers [62] and should be implemented more broadly. However, an overabundance of warning labels could also lead to desensitisation to such warnings [63], reducing their effectiveness. Therefore, a combined approach in which a minimum percentage of healthy products is required would be ideal. Such approaches could be supported through policies such as minimum stocking standards [64].

The lowest-level leverage point that could be used is adjusting the constants, parameters, and numbers of the system (*leverage point 12*): Studies have shown that unhealthy ingredient contents (e.g. sugar) can be reduced substantially with little impact on sales [65], which would substantially benefit public health [66]. Furthermore, adjustments to pricing (e.g. sugar-based taxes) also show promising effects in a recent systematic review [67]. These leverage points have shown promising effects in the USA [65–67], Europe, Scandinavia, Africa, and various other regions [67] and should be expanded upon. However, it should be clear that these are meant as a first step towards addressing the higher-level leverage points outlined above.

### Policy implications

The commercial food system is complex, and the results of this study showed that an availability imbalance between healthy and unhealthy foods is deeply ingrained in how the system operates (i.e. goals, rules, strong reinforcing loops). To resolve this, we recommend a combination of policy actions aimed at higher-level leverage points, to address the core drivers of the problem, and lower-level leverage points, to limit short-term damage to public health and help build momentum for higher-level actions:

- Higher level: Facilitate alternative corporate governance models (e.g. create legal frameworks for such models) that facilitate sustainable business practices, such as steward ownership [51]
- Intermediate level: Explore policy strategies to reduce the restrictive influence of competition on the production and retail of healthier products, for example by establishing a legal baseline or ‘level playing field’, similar to suggestions regarding alcohol marketing [68]
- Lower level: Maintain and expand incentives for the development, reformulation, production, and retail of healthy products, while disincentivising that of unhealthy ones, for example via subsidies or taxes [67]

Although the study was conducted in The Netherlands, its findings are relevant to other high-income countries for several reasons: (i) Various participants represented multinational companies, who likely operate similarly across countries, or academics with expertise beyond the Netherlands; (ii) supermarkets are prevalent in many high-income countries [21], likely operating on comparable principles; (iii) food store environments are dominated by unhealthy products in many countries [5–8] and likely driven by similar dynamics.

### Further research

We have several recommendations for future research. The system is highly complex and our highest-level solution (economic reform) is far removed from the current situation. Research should be done on how to realise such changes, possibly based on transition theory (a discipline specialised in understanding, facilitating, and guiding system change) [69]. It also would be valuable to explore alternative models of food production and retail, where health and commercial goals can stand on an equal level [49, 50]. Furthermore, competition, profit maximisation, and shareholder interests seem to drive the dominant availability of unhealthy products. Future research should explore these drivers in further depth, for example through a commercial determinants of health lens [70]. Finally, the current CLD makes limited distinction between highly processed and less processed food products. The role of power differences between system actors remains underexplored. More research into these topics and how they affect the dynamics at play would be beneficial.

### Strengths and limitations

The study had several limitations, including the use of interviews as main input for the CLD, whereas group model building produces ‘higher-quality models’ [71]. This limitation was accepted to allow participants to speak more freely on commercially sensitive matters. It remains possible that participants refrained from sharing certain sensitive information, and therefore the CLD may be missing important dynamics. Furthermore, because no interview-protocols were published for developing CLDs at the time of data collection, our protocol was based on practices from social sciences instead. Notably, this resembles a recently published interview-protocol for developing CLDs [72], validating our approach. Finally, the study did only examine the Dutch context, although its findings can be argued to be relevant to other high-income countries (see Policy implications).

The study also had several strengths, namely (i) the inclusion of a wide range of system stakeholders and academic experts, to include a variety of perspectives and limiting bias; (ii) the feed-back-feed-forward approach to interviews which allowed interviewees to validate each other’s input; and (iii) triangulation of the data analysis between two researchers to strengthen internal validity.

## Conclusions

Our findings illustrate the complex drivers of the dominant availability of unhealthy food products in supermarket environments. The dynamics of these drivers often span across multiple domains, including the development, production, retail, and consumption of food. Many of these drivers in some way interact with the commercial interests of the involved actors and the value that a product can provide to these interests. Due to a combination of self-reinforcing perceptions regarding consumer preferences and behaviours, lock-in on previously chosen avenues of competition, and risk-aversion in a highly competitive market, unhealthy products generally appear to better align with these commercial interests. As a result, these products tend to be developed, produced, and retailed more often, and therefore are more available. There are several dynamics which facilitate greater availability for healthier products, but these are limited by diminishing return, perceived to negatively interact with commercial outcomes, or specific to niche consumer groups. Major changes through policy and governance will be required to change the incentives and structures of our food development, production, and retail systems to reach a healthier balance between healthy and unhealthy food availability in supermarkets.

## Supplementary Information

Additional file 1: Supplementary File A COREQ checklist; a filled-in checklist for the ‘consolidated criteria for reporting qualitative research’.

Additional file 2: Supplementary File B Interview guide; an English translation of the interview guide used for the interviews.

Additional file 3: Supplementary File C Preliminary CLD; the preliminary version of the CLD, used as input for the focus group with academic experts.

Additional file 4: Supplementary File D Preliminary codebook; the preliminary codebook based on the sketch CLD, used as a starting point for coding the interviews.

## Data Availability

The transcripts used for his study cannot be made publicly available due the sensitive nature of some of the information within. A curated overview of the coded segments is available from the corresponding author on reasonable request.

## Abbreviation

CLD: Causal loop diagram
