# Supplementary material for: Causal loop diagramming the dynamics that shape food environments in Dutch supermarkets
Source: BMC Med. 2025 Oct 23;23:578. doi: 10.1186/s12916-025-04360-z (PMC12548238; doi:10.1186/s12916-025-04360-z)
Supplement: Supplementary file 2 — Additional file 2: Supplementary File B Interview guide; an English translation of the interview guide used for the interviews. [file 12916_2025_4360_MOESM2_ESM.pdf]

Interview Guide

1. Introduction

- The interviewer introduces themselves.
- The participant introduces themselves.
  - "What is the role of your organisation within the food system?"
  - "How does your organisation interact with supermarkets (and their product offer)?"
  - "What is your role in these interactions?"
  - "How long have you been doing this, and what other experience do you have in this sector?"

Explain the purpose and nature of the research:

  - "Our aim is to gain insight into the actors, factors, and dynamics that determine which products are available in supermarkets. We want to visualise these in a type of flow diagram."
  - "We are investigating this by speaking with all the major stakeholders in the Dutch food system."

- Give the participant the opportunity to ask questions.
- Emphasise that the participant may stop the interview at any time or designate any information as confidential.
- Check whether the informed consent form has been signed. If not, ask the participant to do so.
- Start the audio recording (announce this verbally).

----

2. Exploration

Follow the topic list below. Work from top to bottom. Use probing questions where necessary to gather more information.

>>>>>>>>>>>>>>>>>>

Topic 1: Influence of the participant's organisation on supermarket product offer

Opening question:

“Within your organisation, which factors and processes influence the product offer in supermarkets, on the basis of which factors, and who is involved?”

Example probing questions:

- “What direct influences are there within your organisation?”
- ”What indirect influences are there within your organisation? ”
- ”Could you describe this factor/process in more detail?”
- ”What considerations play a role in this process?”
- ”Which factors play a role in these considerations?”
- “what determines this factor?”
- ”Who makes these decisions, and where? ”
- ”Have all important factors now been mentioned?”

>>>>>>>>>>>>>>>>>

Topic 2: External influences on supermarket product offer

Opening question:

“We discussed several processes within your organisation that influence the supermarket product offer. But what processes outside your organisation also have an influence?”

Example probing questions:

- *”Which external processes or factors influence the factors or processes within your organisation? ”*
- *”Do you know of processes or factors do you know of that influence the supermarket product offer, unrelated to your organisation? ”*
- *”Could you describe this factor/process in more detail?”*
- *”What considerations play a role in this process? ”*
- *”Which factors play a role in these considerations? ”*
- *“what determines this factor?”*
- *”Who makes these decisions, and where? ”*

- *”Have all important factors now been mentioned? ”*

>>>>>>>>>>>>>>>>>>

Topic 3: Influences from previous interviews (optional)

Opening question:

“In a previous interview, […] was mentioned as an influence. What is your view on this?”

Example probing questions:

- *”Do you agree with this information? ”*
- *”Would you like to add anything? ”*

>>>>>>>>>>>>>>>>>>

Topic 4: Influence on the availability of healthy/unhealthy products

Opening question:

“What do you think is the effect of the influences we have discussed on the availability of healthy versus unhealthy products?”

Example probing questions:

- *”Why do you think that? ”*
- *”What do you think is the effect of this particular factor? ”*

>>>>>>>>>>>>>>>>>>

Topic 5: Other information

Opening question:

“Are there any important matters we have not yet discussed?”

---

3. Evaluation

Show the participant the CLD diagram containing the actors, factors, and dynamics. Their own answers are not yet included.

- *“Here you see a diagram with the answers from our previous participants.”*
- *“In addition to what we have just discussed, would you like to add or adjust anything on this diagram?”*

If the participant wants to adjust something:

- *"Why do you disagree with this information?"*

If the participant wants to add something:

- *"Why do you consider this information important to add?"*
- *"In your view, given these factors and dynamics, why is it that the supermarket product offer is largely unhealthy?"*
- *"Which of these factors and dynamics play the biggest role? Why?"*

Check whether the participant is satisfied, and if so, conclude this part of the interview.

---

4. Closing

- State that the interview is finished. Stop the recording.
- Briefly explain what will happen with the recording:
    - *"The recording will be encrypted with a password and stored on a secure server. We will create a transcript of the recording, which will also be encrypted and stored on the secure server. We will then make an anonymised version of the transcript, from which all identifying information will be removed, and this will be used for analysis. Based on this analysis, we will write a scientific article."*
- Remind the participant that they can still mark any information as confidential.
- Ask whether the participant has any further questions.
- Ask whether it would be acceptable to contact them at a later stage with additional questions.
- Thank the participant for their participation.

[End of interview]
