# Supplementary material for: Causal loop diagramming the dynamics that shape food environments in Dutch supermarkets
Source: BMC Med. 2025 Oct 23;23:578. doi: 10.1186/s12916-025-04360-z (PMC12548238; doi:10.1186/s12916-025-04360-z)
Supplement: Supplementary file 3 — Additional file 3: Supplementary File C Preliminary CLD; the preliminary version of the CLD, used as input for the focus group with academic experts. [file 12916_2025_4360_MOESM3_ESM.pdf]

Supplementary File D: Preliminary CLD

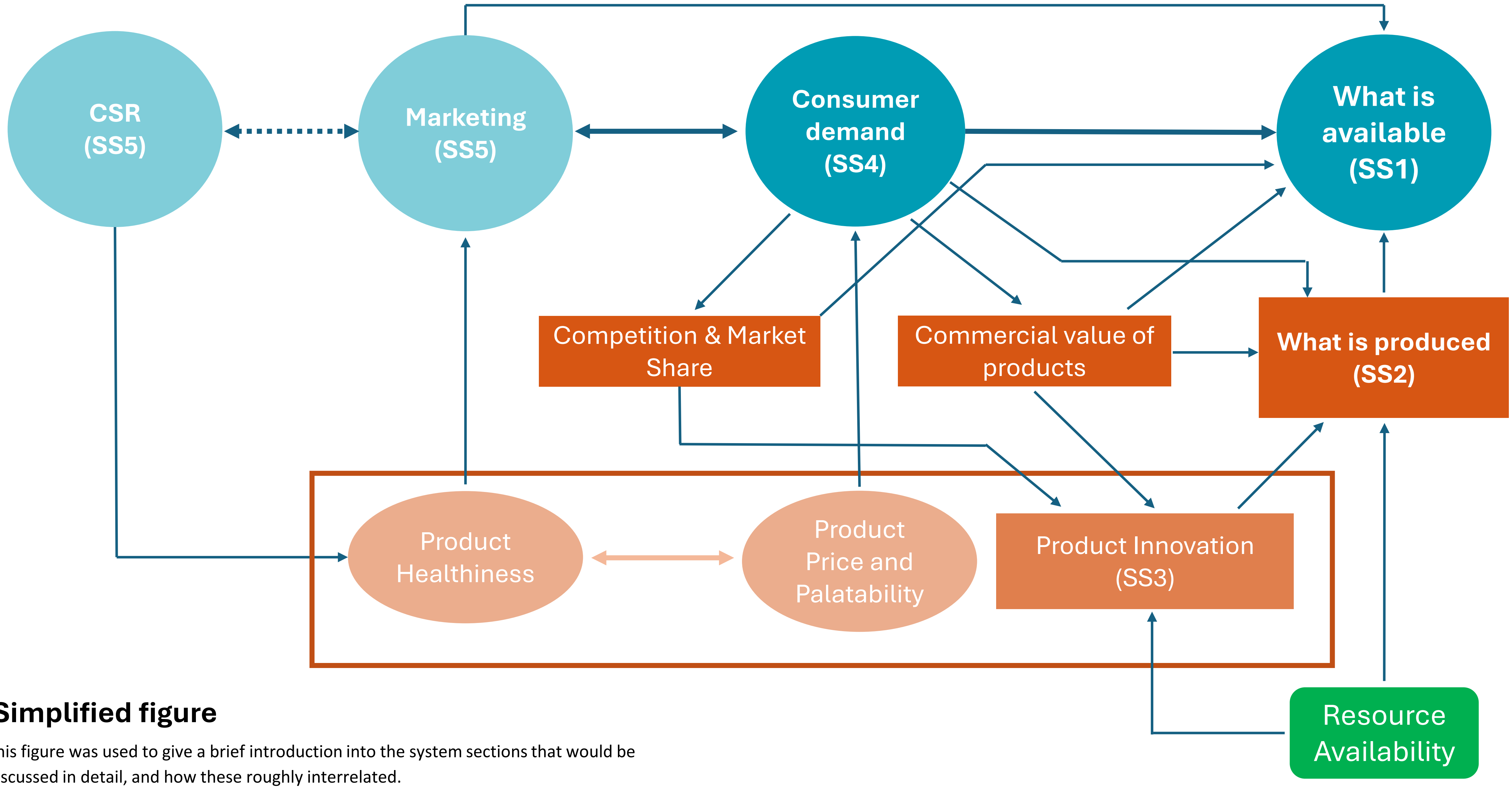

Simplified figure

This figure was used to give a brief introduction into the system sections that would be discussed in detail, and how these roughly interrelated.

Supplementary File D: Preliminary CLD

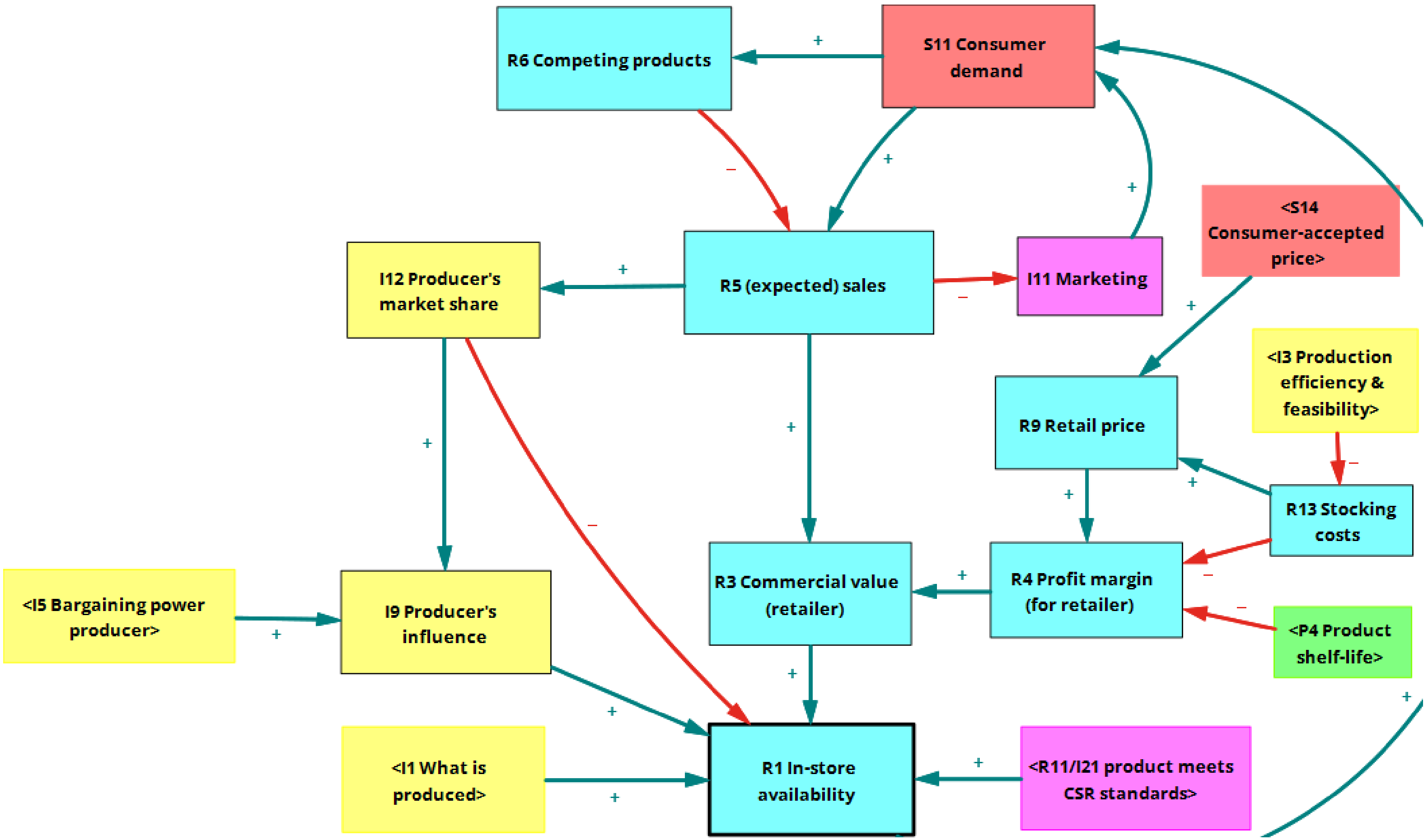

System section 1

Supplementary File D: Preliminary CLD

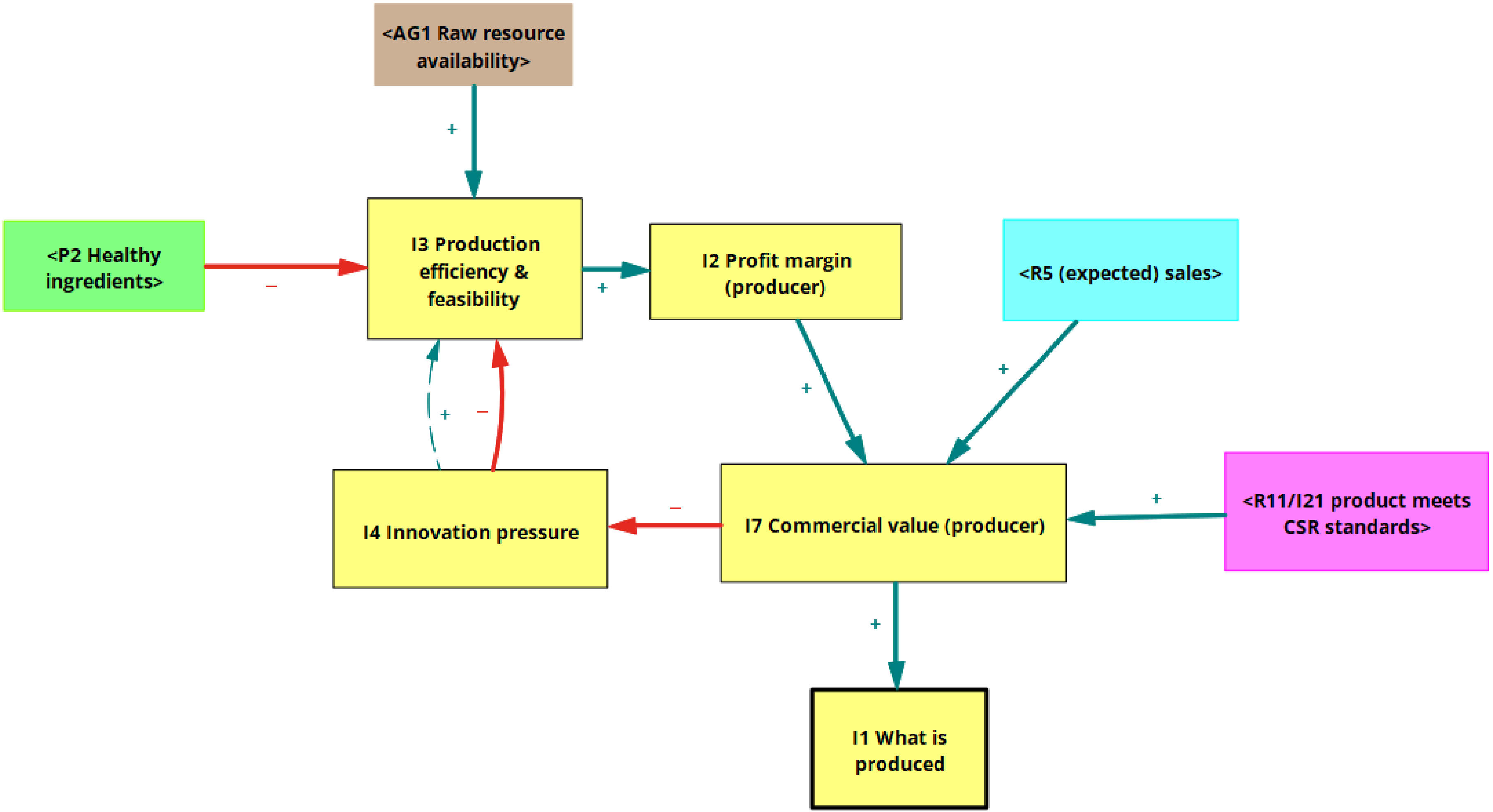

System section 2

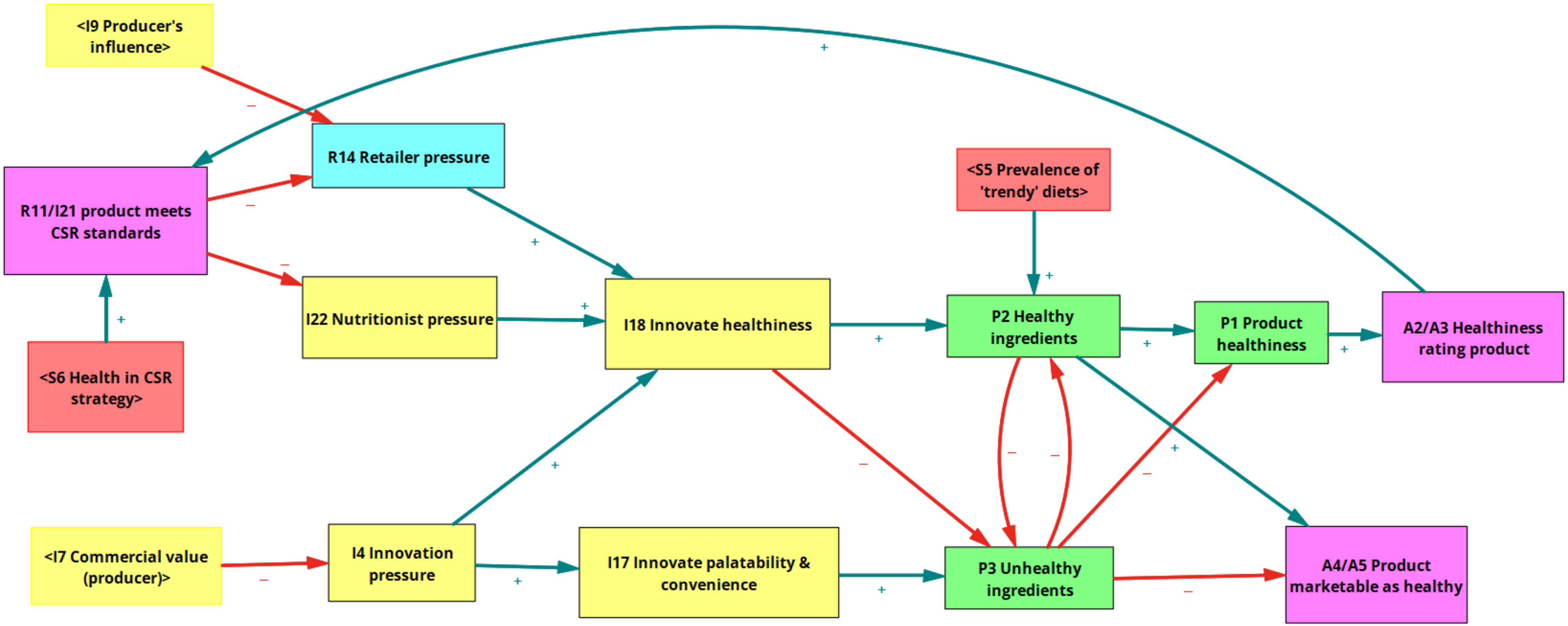

System section 3

Supplementary File D: Preliminary CLD

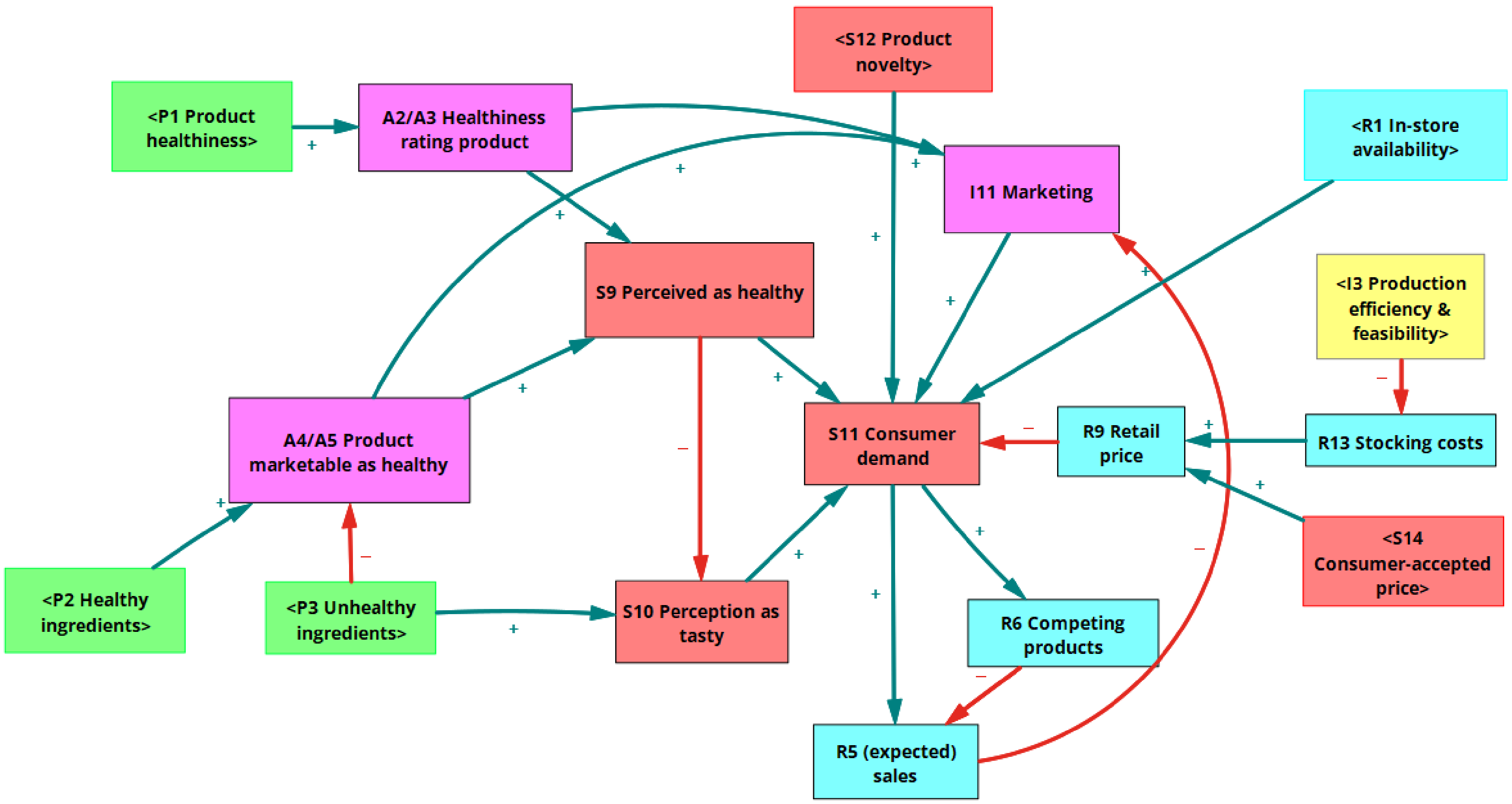

System section 4

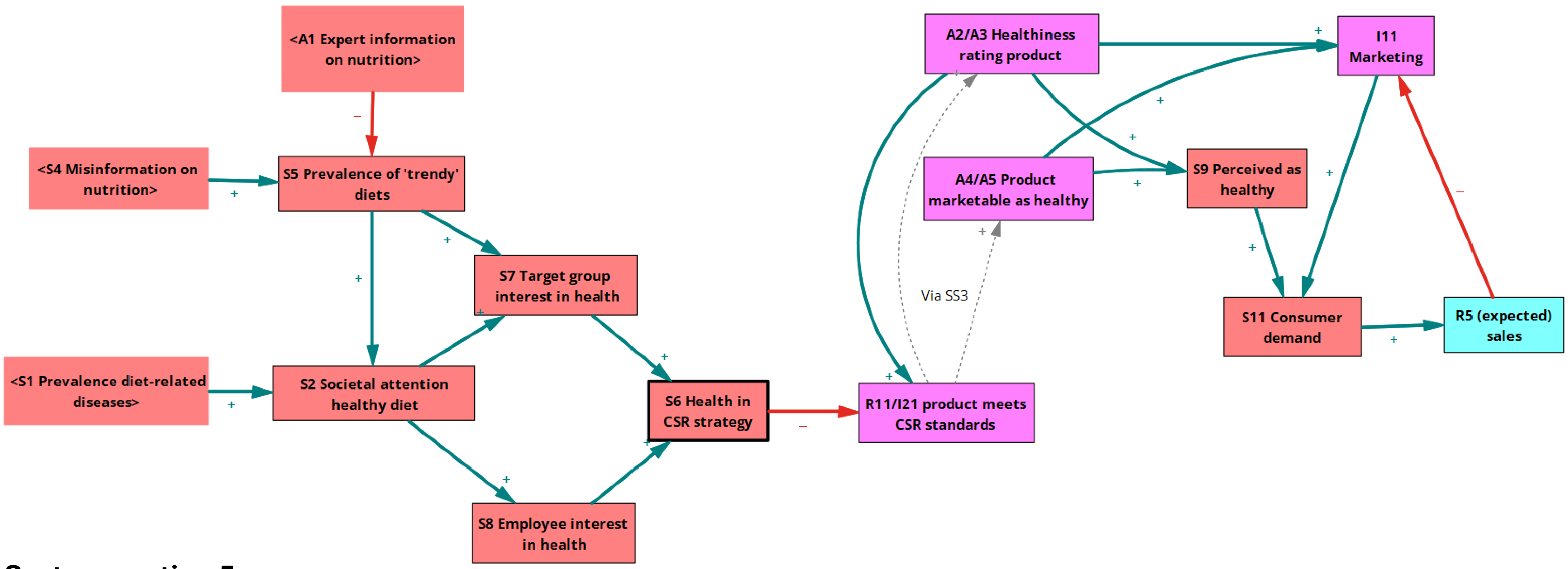

System section 5

Supplementary File D: Preliminary CLD

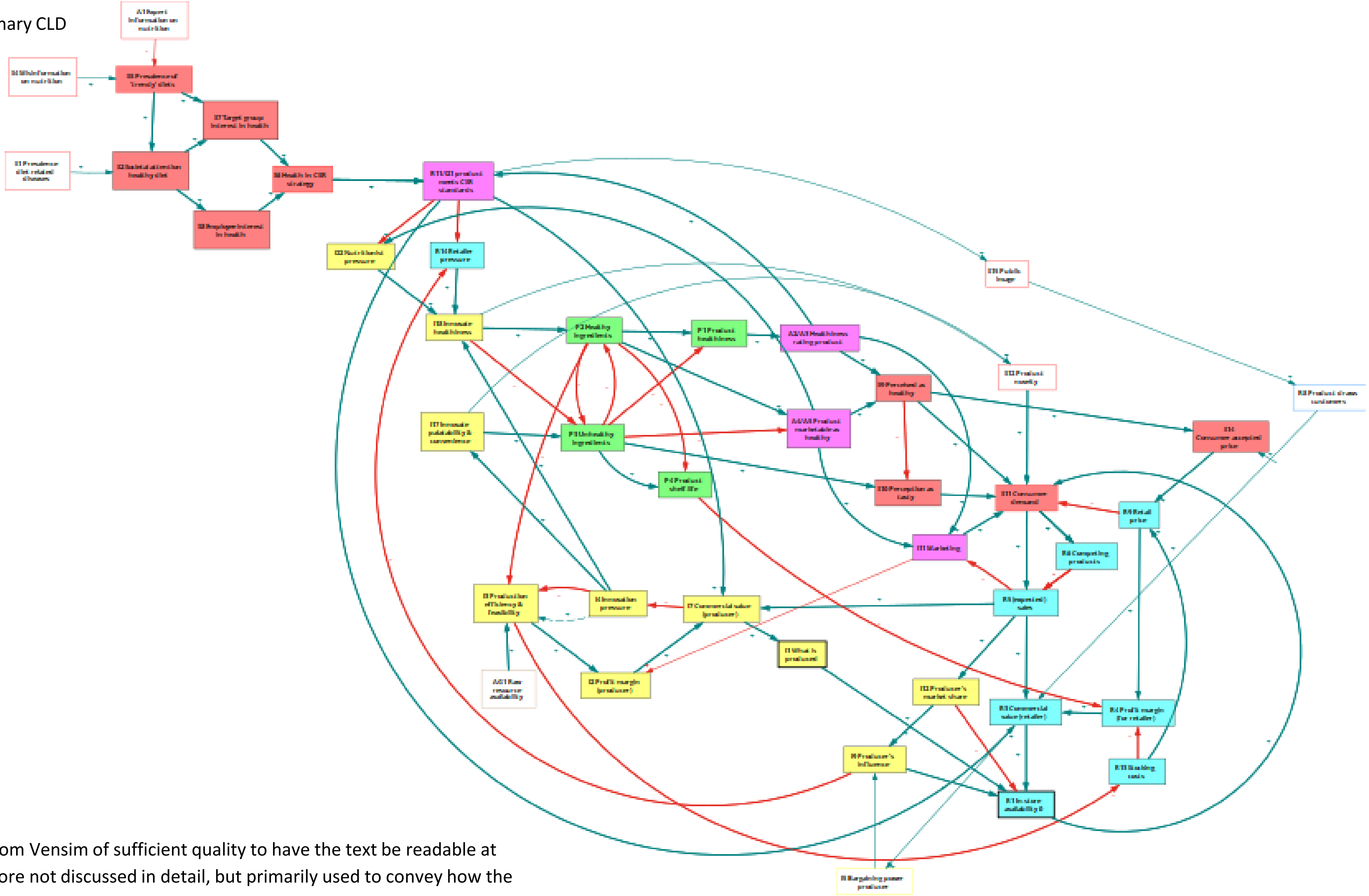

Combined map

We could not export a figure from Vensim of sufficient quality to have the text be readable at this size. This figure was therefore not discussed in detail, but primarily used to convey how the previously discussed section of the system fit together.
